# Cardiac Troponin and Tropomyosin: Structural and Cellular Perspectives to Unveil the Hypertrophic Cardiomyopathy Phenotype

**DOI:** 10.3389/fphys.2016.00429

**Published:** 2016-09-23

**Authors:** Mayra de A. Marques, Guilherme A. P. de Oliveira

**Affiliations:** Programa de Biologia Estrutural, Centro Nacional de Ressonância Magnética Nuclear Jiri Jonas, Instituto de Bioquímica Médica Leopoldo de Meis, Instituto Nacional de Biologia Estrutural e Bioimagem, Universidade Federal do Rio de JaneiroRio de Janeiro, Brazil

**Keywords:** hypertrophic cardiomyopathy, protein dynamics, sarcomeric mutations, thin filament, allostery

## Abstract

Inherited myopathies affect both skeletal and cardiac muscle and are commonly associated with genetic dysfunctions, leading to the production of anomalous proteins. In cardiomyopathies, mutations frequently occur in sarcomeric genes, but the cause-effect scenario between genetic alterations and pathological processes remains elusive. Hypertrophic cardiomyopathy (HCM) was the first cardiac disease associated with a genetic background. Since the discovery of the first mutation in the β-myosin heavy chain, more than 1400 new mutations in 11 sarcomeric genes have been reported, awarding HCM the title of the “disease of the sarcomere.” The most common macroscopic phenotypes are left ventricle and interventricular septal thickening, but because the clinical profile of this disease is quite heterogeneous, these phenotypes are not suitable for an accurate diagnosis. The development of genomic approaches for clinical investigation allows for diagnostic progress and understanding at the molecular level. Meanwhile, the lack of accurate *in vivo* models to better comprehend the cellular events triggered by this pathology has become a challenge. Notwithstanding, the imbalance of Ca^2+^ concentrations, altered signaling pathways, induction of apoptotic factors, and heart remodeling leading to abnormal anatomy have already been reported. Of note, a misbalance of signaling biomolecules, such as kinases and tumor suppressors (e.g., Akt and p53), seems to participate in apoptotic and fibrotic events. In HCM, structural and cellular information about defective sarcomeric proteins and their altered interactome is emerging but still represents a bottleneck for developing new concepts in basic research and for future therapeutic interventions. This review focuses on the structural and cellular alterations triggered by HCM-causing mutations in troponin and tropomyosin proteins and how structural biology can aid in the discovery of new platforms for therapeutics. We highlight the importance of a better understanding of allosteric communications within these thin-filament proteins to decipher the HCM pathological state.

## Introduction

Cardiomyopathies represent a collection of disorders that originate in the heart muscle itself or as a side effect of some other systemic conditions, leading to heart damage and electrical function impairment (Maron et al., 2006). According to the American Heart Association (AHA), cardiomyopathies are classified into two major groups: primary, referring to those predominantly affecting the heart muscle, and secondary, referring to those with the pathological involvement of the heart in a large number of systemic diseases (Maron et al., 2006). Hypertrophic cardiomyopathy (HCM) is a primary muscle disease and the most common cause of sudden cardiovascular death in young athletes; however, cardiovascular death caused by HCM is 8 times more frequent when considering not only young athletes. The same pattern is true for the incidence of HCM, which is 3 times higher in the young population (Maron et al., 2016a). Of note, HCM affects 1 in 500 individuals in the general population, but this ratio may be underestimated due to the lack of information regarding familial cases or asymptomatic subjects (Mozaffarian et al., 2016). HCM was the first cardiac disease associated with a genetic background and presents an autosomal dominant pattern of inheritance. Sequencing efforts have allowed the discovery of the first HCM-causing mutation in the β-myosin heavy chain (*MHY7* gene, R403Q) (Geisterfer-Lowrance et al., 1990). Since then, more than 1400 mutations in 11 sarcomeric genes have been unveiled, and due to this pattern of affected genes, HCM is also called the disease of the sarcomere (Watkins et al., 1992, 1995; Thierfelder et al., 1994; Seidman and Seidman, 2001; Konno et al., 2010).

The clinical profile of HCM is quite heterogeneous. While some patients exhibit severe to mild manifestations, others are completely unaware of having the disease. The initial suspicious of HCM come from a heart murmur during physical activity, family history, or an abnormal echocardiogram (ECG) pattern (Marian, 2010; Maron et al., 2012; Maron and Maron, 2013). Its diagnosis is based on two-dimensional echocardiography, which permits the detection of an asymmetric hypertrophied left ventricle chamber. Of note, the HCM diagnose should be taken in the absence of other diseases with similar clinical profiles (e.g., aortic stenosis or hypertension) (Maron et al., 2014, 2016b). Moreover, other HCM clinical manifestations include left ventricular hypercontractility, cardiac insufficiency, ventricular fibrillation, syncope and arrhythmias. Regarding its morphological and histological features, left ventricle wall and ventricular septum thickening typically occurs (Teare, 1958; Maron et al., 1979; Varnava et al., 2001). The architecture of the hypertrophic myocardial fibers differs in shape and angle arrangement, leading to a chaotic environment (Maron et al., 1981). In combination with cellular disarray, fibrosis with an abnormal collagen matrix is also observed (St. John Sutton et al., 1980; Shirani et al., 2000; Kwon et al., 2009; Nakamura et al., 2016). Indeed, a possible clinical correlation between these findings and HCM pathology impairs the proper relaxation of the heart, preventing it from filling correctly. Damage to the electrical signal conduction may also occur, leading to arrhythmia, tachycardia and ventricular fibrillation, which may ultimately contribute to the development of secondary pathologies, e.g., ischemia or hypotension (Kon-No et al., 2001; Christiaans et al., 2009; Lan et al., 2013; Crocini et al., 2016). Altered ion channels including at least six susceptible genes, e.g., *KVLQT1, HERG, SCN5A, minK, MiRP1*, and *RyR2* play critical steps during the development of arrhythmia phenotypes (Keating and Sanguinetti, 2001). Of note, the ryanodine channel (*RyR2*) triggers the release of Ca^2+^ from the sarcoplasmic reticulum to start contraction. Mutations in *RyR2* lead to aberrant intracellular Ca^2+^ metabolism and Ca^2+^ overload that may have an involvement in arrhythmias (Keating and Sanguinetti, 2001). Additionally, during the phase 0 depolarization of the cardiac action potential, the binding of calmodulin to the C-terminal region of the hH1 Na^+^ channel occurs in a Ca^2+^-dependent manner and impact the slow inactivation gating process with implications to cardiac arrhythmias (Tan et al., 2002). Because the HCM clinical phenotype ranges from asymptomatic subjects to patients who require surgery or transplant, it is reasonable to use both clinical data and imaging tests during initial screening, but this may not be the most effective approach for the diagnosis probands carrying a silent disease. Genetic tests are available for molecular diagnosis, to identify HCM-causing mutations of the proband and for family screening (Ho et al., 2015). These trials were conducted at the bench and were breakthroughs, promoting a fast and reliable diagnosis (Maron et al., 2012). However, despite all efforts in the molecular biology field, the association between mutational profile and disease phenotype remains elusive. For instance, one interesting question is why some mutations trigger a pathogenic status while others lead to a benign course. The most parsimonious explanation is that mutations not only affect protein function but may also cause changes in folding, dynamics and interactomes. In this review, we focused on recent discussions of the structural basis for the effects and cellular consequences of HCM mutations.

## From code to message

Almost 50 years ago, Francis Crick published a noteworthy article entitled the “Central dogma of molecular biology” (Crick, 1970). At that time, it was postulated that in the cellular environment, the flow of information initiates through the deoxyribonucleic acid (DNA) molecule that contains all of the elements for protein production. The intermediate molecule responsible for carrying a copy of the target protein is called ribonucleic acid (RNA). Finally, this template requires a complex cellular machinery for protein synthesis.

**Figure 1:**
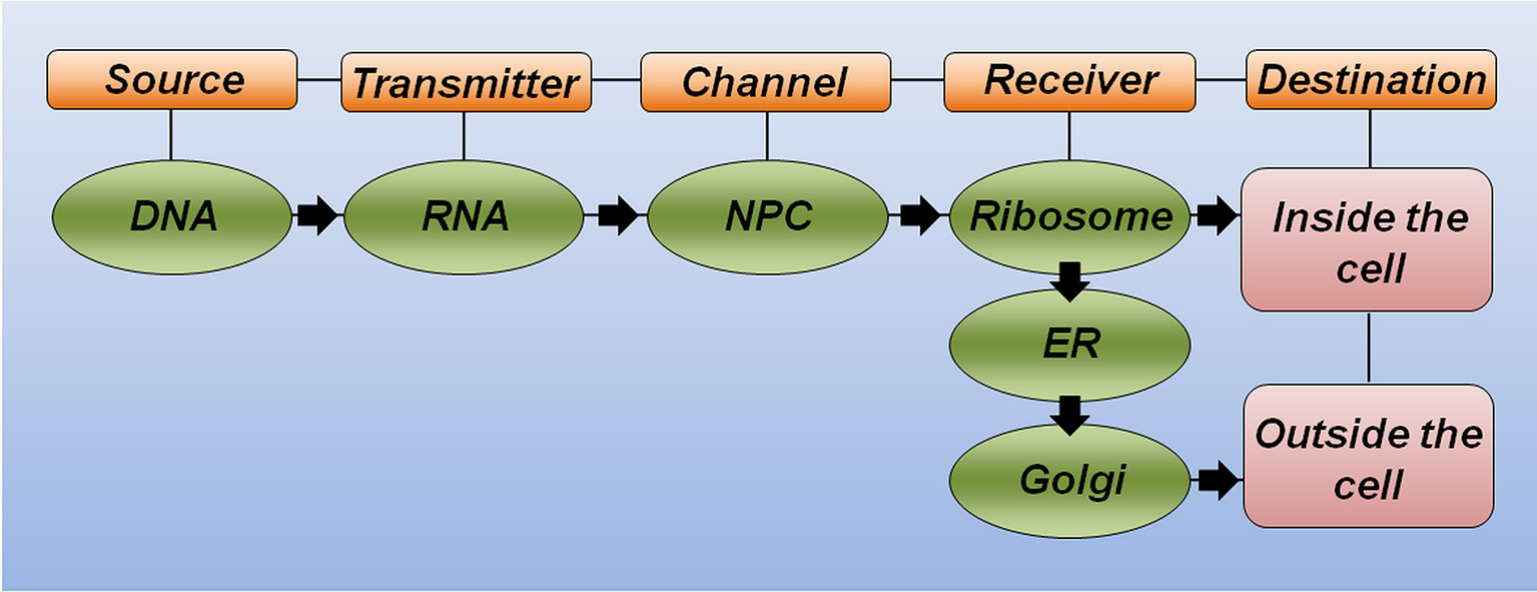
**The theory of communication in biology**. Schematic representation highlighting the key elements for correct communication in biology. DNA is the source of information and carries the correct message to produce a functional protein. RNA molecules link the source to the channel. The nuclear pore complex (NPC) serves as a selective channel and sends the RNA to the receiver. The ribosome is the first receiver responsible for making the message intelligible (translate from RNA to protein). Additional receivers, including the endoplasmatic reticulum (ER) and the Golgi apparatus, will help the protein to achieve its proper folding and post-translational modification to reach the final destination and correct response.

Drawing a parallel between the central dogma of molecular biology and the theory of communication (Figure 1), five key elements should be considered for successful communication. (i) The *source of information* is important for producing the message. An analogy of the source could be a sequence of letters organized in a coherent way to form a word or sentence. (ii) The *transmitter* is responsible for sending this message through a channel. (iii) The *channel* links the transmitter to the receiver. (iv) The *receiver* makes the message intelligible when reaching its destination. (v) The *destination* is where the message arrives and can then perform its function (Shannon, 1948).

Considering the polypeptide chain of a hypothetical protein as a message to be sent, the specific DNA region that carries the correct nucleotide sequence for this protein (i.e., the gene) serves as the source of information. Equally important, the RNA molecule plays the transmitter role, allowing the correct message to reach the channel. The nuclear pore complex recognizes mature RNA molecules and sends them to the receiver, thus serving as a selective channel. Next, the complex machinery of the ribosome acts as the receiver, transforming the genetic information through the synthesis of a correct polypeptide sequence. In this analogy, additional receivers would allow proper polypeptide folding (e.g., the endoplasmatic reticulum and chaperone molecules) and the incorporation of important modifications, such as phosphorylation and glycosylation, among others (e.g., kinase proteins and the Golgi apparatus). Finally, the destination of this functional protein will generate the expected response inside or outside the cell. This simplistic analogy illustrates how any substitution occurring at the message level may significantly affect the correct communication and cause either a misunderstanding or a new understanding at the destination (Figure 1).

Correct folding is intimately linked to the function or, analogously, the message of a biomolecule. A remarkable study in the 1950s showed that the three-dimensional assembly of a protein is guided by its specific amino acid sequence. The physicochemical properties of specific amino acid side chains lead to a hydrophobic collapse event; thus, proper folding is expected to occur independently of biological cellular machinery (Anfinsen et al., 1961; Anfinsen, 1973). The energy landscape is an accepted theory to explain the protein folding phenomenon (Onuchic et al., 1997). Proteins normally experience a wide range of conformational changes to reach their low-energy native state. In terms of free energy, folding pathways are commonly summarized using schematic funnels, in which high-energy protein stages (i.e., denatured polypeptides) are guided through preferential intramolecular contacts to achieve the lowest free energy and conformational entropy (i.e., native polypeptides). The folding of a single polypeptide chain, therefore, occurs through the assembly of partially folded intermediates due to dynamics and, together with water solvation, plays an essential role in this process (Bai and Englander, 1996; Dill and Chan, 1997; Onuchic, 1997; Cheung et al., 2002; Onuchic and Wolynes, 2004; Dill et al., 2007).

**Figure 2:**
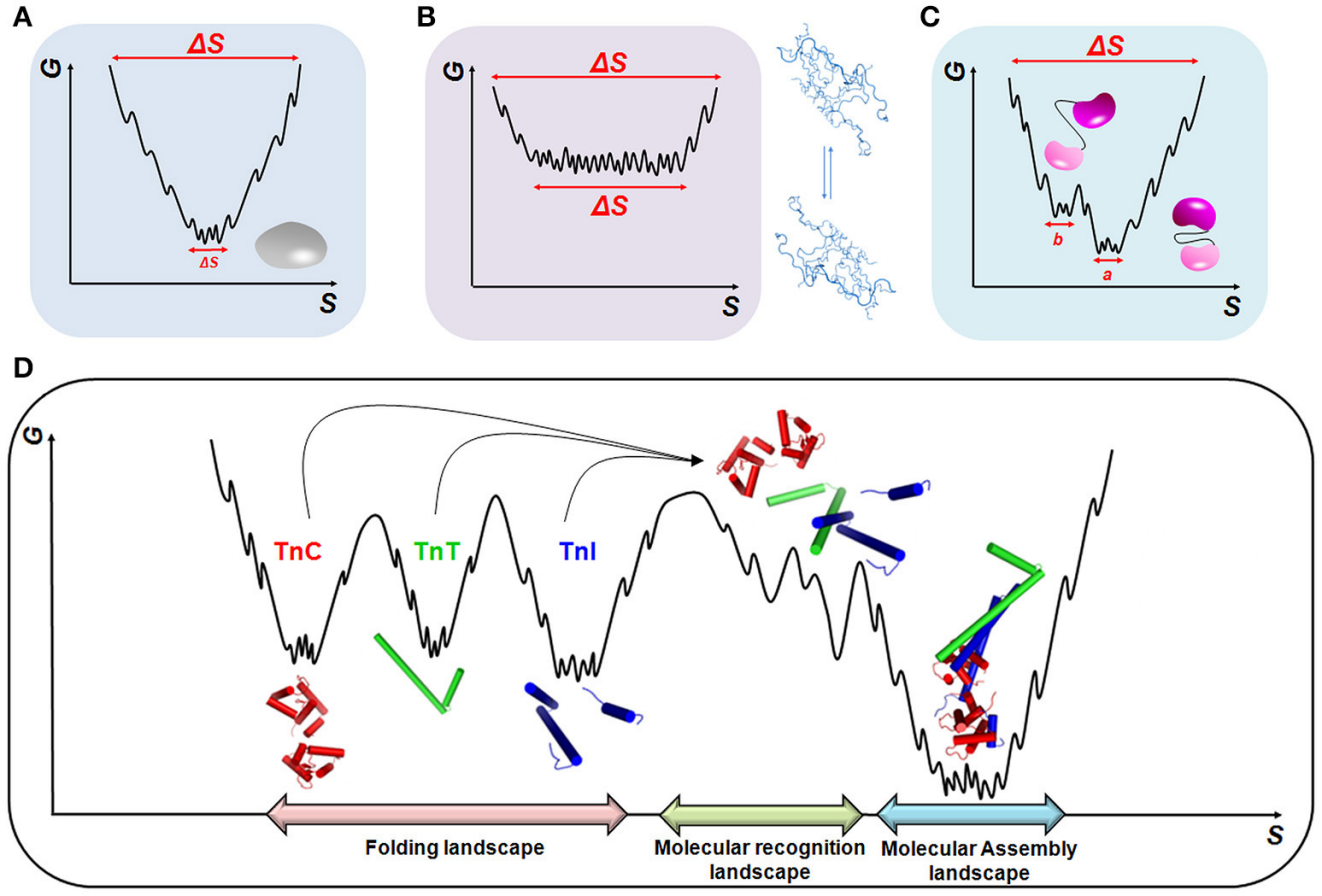
**Energetic landscapes**. Schematic representing the minimization of conformational entropy (*S*) and free energy (*G*) during the folding of **(A)** globular proteins, **(B)** intrinsically disordered proteins (IDPs), and **(C)** multi-domain proteins. In **(C)**, two different conformations, *a* and *b*, triggered by linker motions, are represented. **(D)** Molecular recognition and assembly landscapes for the cTn complex formed by cTnC, cTnI, and cTnT (PDB code 1j1e). During molecular recognition, higher-energy sub-states are formed to guarantee the correct sampling among the different biological partners.

The assembly of tightly bound or transiently bound molecular complexes in the perspective of energetic landscapes requires an orchestrated and hierarchic environment in which a range of molecular motions take place (Figure 2). Polypeptide chains are intrinsically dynamic entities sampling different conformations from pico- to millisecond timescales during folding and upon activation or molecular recognition. Rapid fluctuations (i.e., thermal motions on the order of 10^−12^–10^−9^ s) commonly occur in native globular proteins and result in structurally similar conformers with implications for molecular recognition (Figure 2A). At the opposite side of the energetic landscape, intrinsically disordered proteins (IDPs) are very flexible molecular motors, sampling heterogeneous conformations with high free energy and conformational entropy (Figure 2B). In both scenarios, we may exemplify slower timescale dynamics (i.e., on the order of 10^−6^–10^−3^ s) in which a small number of high-energy and short-lived conformers are populated (Figure 2C). This condition is normally observed in more complex proteins with intrinsically disordered regions (IDRs), such as flexible linkers or hinge-connecting domains, and that participate in relevant biological processes, including molecular assemblies, catalysis and ion-coordination. In the context of multi-domain proteins (i.e., globular domains linked by IDRs) and the broad spectrum of motions that arise from these complexes (Figure 2D), an understanding of how hierarchical motions trigger the formation of tightly or transiently bound molecular assemblies and the physiological and pathological consequences is just now emerging.

The consequences of alterations in the DNA can be compared to “*heaven or hell.*” Evolutionary events are commonly beneficial in which adaptive species and precise protein functionalities are selected but may also be catastrophic when loss-of-function (LoF) or gain-of-function (GoF) events occur. Upon mutation, LoF and GoF events are commonly observed in different proteins, including tumor suppressors and sarcomeric proteins, with implications in cancer and cardiac disorders (Marston et al., 2013; Silva et al., 2014). Several organisms have developed specialized repair machineries to address DNA variations and avoid disease, but this is not an infallible process. In addition, the evolution of polypeptide chains does not always result in the correct alphabet sequence (Goldschmidt et al., 2010; Eichner and Radford, 2011). The consequence is that protein misfolding is attributed to more than 50 diseases (Chiti and Dobson, 2006). DNA alterations can change the phenotype with positive, negative or neutral consequences to the adaptability of individuals. The production of faulty proteins causes functional impairment, which may alter contacts with molecular partners, leading to dramatic cellular responses. Among the main variations observed in the DNA, one type can be highlighted in the context of cardiomyopathies: single point mutations. Approximately 90% of these pathogenic mutations are missense, in which one amino acid is changed to another, leading to abnormal molecular, functional and physical properties of the heart.

Pathogenic mutations have at least two important features: (i) they lead to alterations in protein structure and function and (ii) changes frequently occur in highly conserved amino acids throughout evolution (Richards et al., 2008; Maron et al., 2012). In HCM, these two features are observed, and one mutation affecting a conserved region can disrupt pivotal protein-protein interactions, leading to structural chaos. Interestingly, because only key amino acids contribute to the free energy of binding, interaction sites frequently display an asymmetrically energy distribution along the surface. The presence of highly conserved amino acids surrounding the interaction interface plays a key role in stabilizing molecular contacts. Accordingly, complementary pockets comprising bulky side chains tightly stabilize the interaction in a geometrically and energetically favorable manner (Keskin et al., 2005; Metz et al., 2012). When dealing with protein-protein interactions, in addition to size and chemical complementarities, conformational fluctuations in different timescales also take place for proper binding and signaling (Henzler-Wildman and Kern, 2007; Zen et al., 2010). Mutations in highly conserved regions frequently result in steric impairment and conformational changes with LoF or GoF events. Thus, protein dynamics are highly relevant when considering the investigation of protein-protein interactions in physiological and pathological processes.

With regard to RNA modifications impacting cardiomyopathy phenotypes, much attention are being taken to non-conding RNAs (e.g., microRNAs—miRNA) and the process of alternative cleavage and polyadenylation (APA) of mRNAs as regulators of gene expression. For instance, downregulation of a specific miRNA locus in stressed cardiomyocytes is sufficient to attenuate the increase of cell size (Clark et al., 2016) and the levels of miR-499 are increased in failing and hypertrophied hearts with consequences to the levels of target mRNAs. Proteomic analysis linked to miR-499 identified changes in kinase and phosphatase signaling, supporting the key role of these non-conding RNAs in the pathological regulation of cardiomyopathies (Matkovich et al., 2012). Of note, the secretion of miR-29a in the plasma of HCM subjects is associated to both hypertrophy and fibrosis and may serve as a potential biomarker in HCM supporting a direct role of miRNAs in the HCM pathogenesis (Roncarati et al., 2014). Additionally, the APA process of mRNAs is particularly important to generate RNA isoforms and modulate the levels of protein expression in specific genes. In dilated cardiomyopathy a group of genes involved in RNA and actin binding and structural proteins of the cytoskeleton revealed a different profile of mRNA cleavage and polyadenylation and it may account for an additional level of regulation in failing hearts (Creemers et al., 2016).

The bottleneck in our current understanding is the assessment of dynamic changes arising from mutational events in well-organized assemblies, such as the sarcomere, and defects generating pathogenic profiles. More interesting, different mutations in diverse targets can culminate in the same disease but sometimes with distinct phenotypes.

## The sarcomere

To better understand the complexity of the sarcomere, it is important to address some important structural features about this system (Clark et al., 2002; Gautel and Djinović-Carugo, 2016). The sarcomere is a basic contractile unit that repeats regularly throughout myofibrils (Huxley and Niedergerke, 1954) being the responsible for the transformation of chemical energy into mechanical energy, thereby triggering contraction (Bers, 2001). To execute this task, the architecture of the sarcomere is finely orchestrated. Through electron microscopy visualization, several elements can be identified including the Z-discs, M lines, A bands, and H zones. The borders of a sarcomere unit are defined by Z-discs and are the places where the thin filaments, titin and nebulin are anchored. These discs are also involved in mechanosensitivity and nuclear signaling, which contribute to the maintenance of muscle homeostasis (Clark et al., 2002). Of note, the giant titin protein has received much attention with regard to its potential role in the passive and residual force enhancement (Herzog and Leonard, 2002). Stretching active and passive myofibrils to a length that avoided any force contributions from actin-myosin cross-bridges revealed greater force generation in actively when comparing to passively stretched myofibrils, supporting the involvement of titin molecules on this force generation mechanism (Leonard and Herzog, 2010). A three-filament model of force production emerged from these findings with the participation of Ca^2+^ and actin binding to titin molecules (Herzog et al., 2016 and references therein). For more in-depth information on the advantages and limitations of the actin-myosin-titin communication for force generation (three-filament model) with respect to the classical view of cross-bridge, please refer to specialized literature (Herzog et al., 2016; Li et al., 2016).

The M line is the transverse structure located in the center of the sarcomere and has been proposed to be the anchor site of the thick filament through the formation of cross-bridges (Obermann et al., 1996; Agarkova et al., 2003). Toward the center of the sarcomere is the A band composed of thick filaments and associated proteins, e.g., the myosin-binding protein C. This protein plays an important role in regulating myosin polymerization and aligning the thick filaments within the A band. Within this region, a whiteness segment called H is also observed and consists of thin filaments that do not overlap into thick filaments (Clark et al., 2002). The thick filaments are mainly composed of myosin which has three functional domains: the head, the neck, and the tail (Sellers, 2000). The head is the motor domain binding ATP and actin, while the neck region binds to its light chains or calmodulin. Finally, the myosin tail anchors and moves the motor domain toward an efficient interaction with actin (Saez et al., 1987; Sellers, 2000). Six subunits promote a hexameric three-dimensional architecture that includes two heavy chains (myosin heavy chain, MHC) and four light chains (myosin light chain, MLC). The MLC is divided in two domains with regulatory functions and another two domains with structural functions. Together, they finely adjust the motor activity of myosin and the versatility of its kinetics (Milligan, 1996).

The thin filament is the major Ca^2+^ regulation site and comprises actin, tropomyosin (Tm) and the troponin complex (Tn). Actin is a ubiquitously expressed protein and participates in various cellular events, such as motility, cytokinesis and contraction. Although some actin mutations are involved in the HCM (Bai et al., 2015), we will focus this review on the Tn and Tm mutations. Along the length of monomeric actin resides Tm, a “coiled-coil” dimer that interacts in a “head-tail” manner to form a substantial and almost uninterrupted structure around the actin helices. One “coiled” motif interacts with seven actin monomers via saline ionic interactions or through Mg^2+^, and its main function is to inhibit myosin ATPase activity in the absence of Ca^2+^ (Zot and Potter, 1987). The cardiac Tn (cTn) complex is composed of three subunits (C, I, and T) with different three-dimensional structures and functions. Together, these subunits perform the important role of Ca^2+^ and contraction regulation. Cardiac troponin C (cTnC) is the direct Ca^2+^ sensor in the myofilament. The conformational changes triggered by Ca^2+^ binding at specific cTnC sites control the allosteric signaling cascade along the entire complex. Cardiac troponin I (cTnI) performs the classical role of ATPase activity inhibition. In addition, its tridimensional structure plays an important regulatory role in protein-protein interactions. The cardiac troponin T (cTnT) is the “molecular glue” that anchors the Tn members to the thin filament, playing an important role in Ca^2+^ transduction structural signaling. Together, the Tn complex and Tm represent the regulatory proteins of the thin filament (Zot and Potter, 1987).

## The thin filament regulators

The cardiac contraction-relaxation cycle is a physiological event controlled by electric and neurohormonal factors. At the molecular level, this process requires sophisticated protein machinery, i.e., the sarcomere, to manage both chemical and mechanical processes. Particularly interesting, this exquisite machinery exhibits an extensive intra- and intermolecular network. In this context, fine-tuned protein-protein interactions are essential for the correct contraction-relaxation operation. The Tn complex and Tm are the key macromolecules responsible for the modulation of both dynamics and structural signaling along the myofilament. As will be discussed further, these proteins ultimately regulate the exposure of the myosin-actin binding sites.

## Troponin I

**Figure 3:**
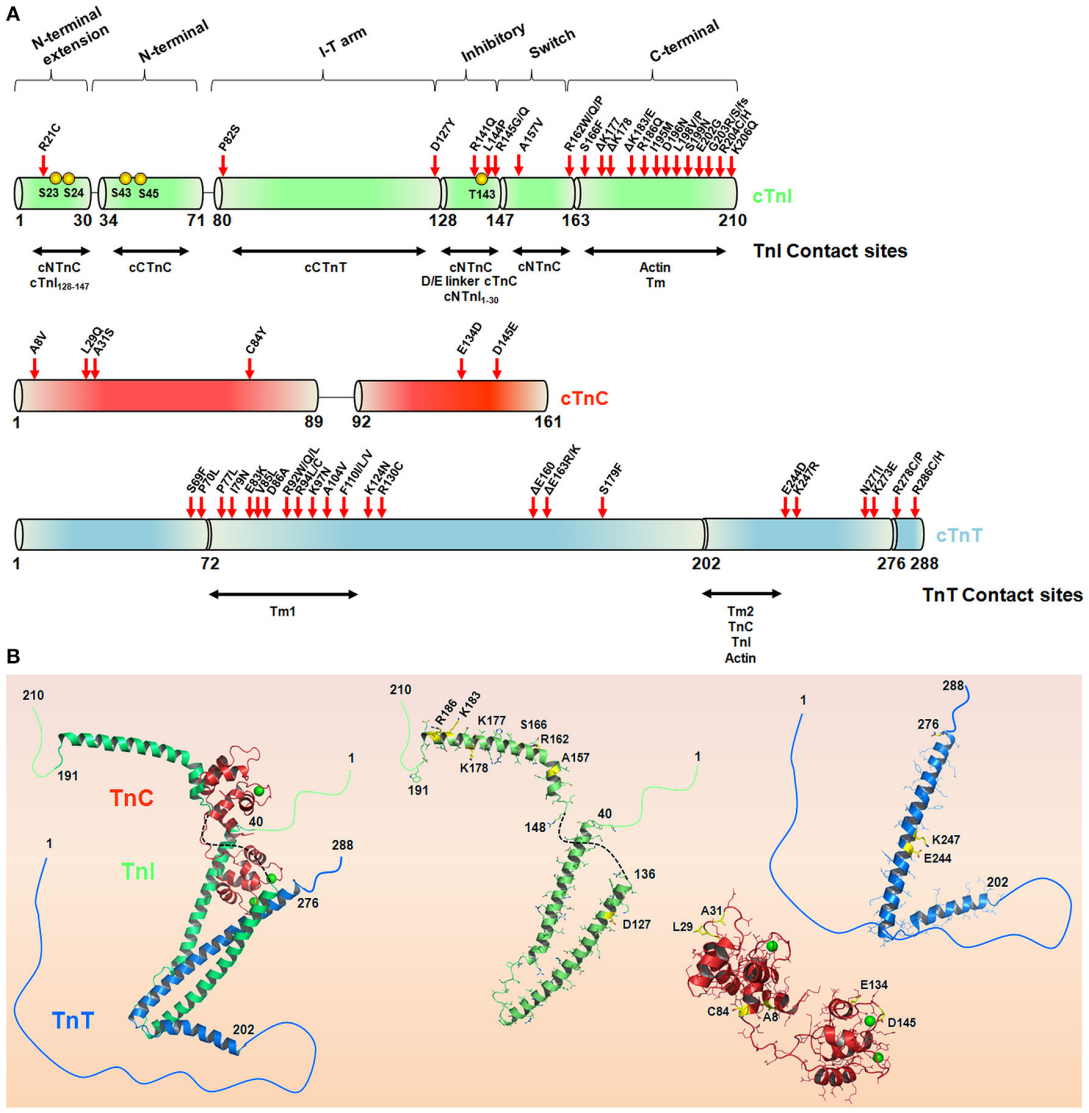
**The hypertrophic cardiomyopathy mutations in the troponin complex. (A)** Schematic representation of the cTnI, cTnC, and cTnT sequences. The TnI and TnT contact sites are depicted in horizontal arrows. Red arrows highlight HCM-causing mutations in cTn subunits in accordance with Willott et al. (2010). Yellow spheres represent cTnI phosphorylation sites. **(B)** Crystal structure of the Tn complex (PDB code 1j1e) and isolated subunits cTnI (green), cTnT (blue), and cTnC (red), highlighting some residues (yellow sticks) affected by HCM-related mutations. The green spheres in cTnC represent Ca^2+^, and the black dashed line represents the inhibitory peptide sequence of cTnI. Note, position 84 in cTnC (PDB code 1j1e) is serine instead of cysteine.

cTnI is the inhibitory unit of the Tn complex and plays an important role as a structural regulator of actomyosin ATPase activity (Leavis and Gergely, 1984). During a heartbeat, cTnI participates in the systolic/diastolic cycle upon changes in the intracellular Ca^2+^ concentration. Using a series of C-terminal mutations, the regulatory segments of TnI involved in the anchoring of TnC to the thin filament start to be addressed (Ramos, 1999). Currently, cTnI can be divided into six different functional regions (Li et al., 2004): (i) an N-terminal extension region (residues 1–30) containing two PKA-dependent phosphorylation sites (Ser23 and Ser24) (Robertson et al., 1982; Chandra et al., 1997); (ii) an N-terminal region (residues 34–71) that interacts with the cTnC C-domain, playing a structural role (Gasmi-Seabrook et al., 1999; Mercier et al., 2000); (iii) a region that binds to cTnT (residues 80–136) as part of the IT-arm; (iv) an inhibitory region (TnI_128−147_) containing a highly conserved amino acid sequence among TnI isoforms; (v) a switch region (cTnI_147−163_) that experiences a disordered-ordered transition upon binding to the N-terminal domain of cTnC-triggering contraction (Li et al., 1999); and (vi) the C-terminal region (residues 164–210) serving as a second consensus site that binds to actin and Tm (Solaro, 2010; Figure 3). Upon β-adrenergic stimulus, two PKA-dependent phosphorylation sites (Ser23/24) within the N-terminal segment of cTnI become phosphorylated and play important roles in the Ca^2+^ desensitization activity of the myofilament, culminating in heart relaxation (Zhang et al., 1995; Solaro et al., 2013). Additionally, other residues in cTnI, including Ser43/45 and Thr143, were shown to be phosphorylated by PKC (Noland et al., 1995), with physiological and pathological implications (Solaro et al., 2013). These N-terminal residues of cardiac TnI are absent in fast and slow TnI isoforms, providing an additional key regulatory segment for the cardiac tissue. At the central region of cTnI, the inhibitory segment (residues 128–147) was shown to attach to actin in the absence of Ca^2+^ but shifts toward an interaction with cTnC upon Ca^2+^ addition (Potter and Gergely, 1974; Kobayashi et al., 1999).

In fact, cTnI is an important molecular switch during the systolic/diastolic cycle. During diastole, low levels of Ca^2+^ stabilize a cTnI conformation that suppresses the power stroke and thus prevents actin-myosin interactions, mostly because the cTnI inhibitory region is sitting on actin (Solaro, 2010). During β-adrenergic stimulation, the intracellular Ca^2+^ concentration increases from a diastolic level of 100 nmol/L to a systolic level of 1 mmol/L, enabling contraction (Bers, 2000). The affinity of cTnI to actin is reduced upon the binding of Ca^2+^ to the N-terminal domain of cTnC (cNTnC), releasing actin inhibition (Solaro and Rarick, 1998). The cTnI inhibitory region now interacts in close proximity to the D/E linker in the cTnC (Lindhout and Sykes, 2003), and the cTnI switch region moves toward the exposed hydrophobic patch in the cNTnC, stabilizing its open (and active) conformation. This mechanism causes cTnI to serve as a molecular latch mediating actin exposure in a Ca^2+^-dependent manner to trigger or inhibit contraction.

Due to the important regulatory role of cTnI in the thin filament, mutations that affect cTn structural cooperativity can lead to the development of diseases. Because several studies have attempted to characterize and better understand the functional defects of cTn mutations in the HCM phenotype, our main focus here is to provide an overview of groundbreaking works and recent literature; unfortunately, we cannot provide a complete review of this vast and enthusiastic literature. Please, for additional studies, see the references cited therein.

The majority of cTnI mutations associated with the HCM phenotype are located in the C-terminal region (Figure 3). Kimura and coworkers reported the first six mutations (R145G, R145Q, R162W, ΔK183, G203S, and K206Q), and almost 30 variations have been reported so far (Kimura et al., 1997; Willott et al., 2010). Interestingly, almost 60% of the HCM-causing cTnI mutations occur through the substitution of a positively or negatively charged amino acid for a neutral or hydrophobic one. Of note, arginine replacement occurs ~40% of the time (Willott et al., 2010). Most of the functional defects triggered by these mutations were explored using skinned cardiac muscle fibers. For example, all of these mutants except for G203S had increased Ca^2+^ sensitivity of myofibrillar ATPase activity and force generation (Takahashi-Yanaga et al., 2001a). As expected, both mutations at position 145 comprising the inhibitory consensus site presented decreased inhibitory cTnI activity, in contrast to R162W and ΔK183, which presented decreased affinity for the cTnI-actin interaction. With the exception of R162W, none of the other mutations perturbed the cTnI-cTnC interaction (Takahashi-Yanaga et al., 2001a). Using surface plasmon resonance, R162W revealed higher affinity for cTnC in the presence of Ca^2+^ (Elliott et al., 2000). Further investigations have explained how R145G impacts the cTnI inhibitory activity. Using skinned cardiac fibers, the authors revealed that this mutation impairs force development and muscle relaxation, possibly explaining some of the clinical features of HCM (Lang et al., 2002). Interestingly, cTnI mutations located at the second actin-Tm-binding site (D190H and R192H) did not increase the levels of ATPase activity, in contrast to the previous R145G mutation located at the cTnI inhibitory segment (Kobayashi and Solaro, 2006). Pathogenic mutations occur at different regulatory segments of cTnI (for the cTnI mutation involved in cardiomyopathies, please Willott et al., 2010). This observation provides insights into the potential repertoire of functional defects that cTnI would be involved. Because cTnI is involved in a series of complex interactions with different biological partners in the thin filament, the expectation is that different cTnI mutation sites will trigger distinct functional defects, as clearly observed in the literature. Although the literature is well designed and ongoing, the explanation of how each of these mutations reflects the clinical HCM phenotypes seems to be challenging.

The development of transgenic animal models represents a serendipitous way to associate mutational defects with disease phenotypes. For example, transgenic mice carrying cTnI R146G (R145G in humans) presented cardiomyocyte disarray, fibrosis, and hypercontractility with diastolic dysfunction, symptoms that align with an increased sensitivity to Ca^2+^ and the HCM phenotype (James et al., 2000). In contrast, a slight decrease in the Ca^2+^ sensitivity of force development was also reported for these cTnI R146G mice (Kruger et al., 2005). In an elegant study, Wen and coauthors were able to study using R245G transgenic mice concomitant measurements of force and actomyosin ATPase activity in skinned papillary fibers to extract the rate of cross-bridge turnover and energy cost. Compared to fibers from a human cTnI wt mice, the fibers from the R245G mice decreased the average force per cross-bridge and revealed a higher energy expenditure, suggesting that in the HCM phenotype, compensatory mechanisms may take place in the heart of R145G mice (Wen et al., 2008). More recently, molecular dynamic (MD) simulations using the whole cTn complex incorporated with cTnI-wt, cTnI-R145G and the cTnI-R145G/S23D/S24D phosphomimetics provided atomistic details to explain new interactions between cNTnC (residues 1–89) and cNTnI (residues 1–41) and the effects of R145G on these interactions (Lindert et al., 2015). The incorporation of aspartic acid substitutions at the PKA-dependent phosphorylation sites of cTnI was validated to recapitulate the same contractile properties and Tn function as does PKA (Lindert et al., 2015 and references therein). The MD simulations revealed that in the cTn complex incorporated with the wt cTnI containing the S23D/S24D phosphomimetics, the loss of contact between cNTnI region 1–41 and the A and B helices of cNTnC occur because of the repositioning of the cNTnI region to interact with the cTnI inhibitory peptide due to phosphorylation. Accordingly, this disordered N-terminal segment of cTnI was previously shown to interact with the cNTnC in the unphosphorylated state, thereby stabilizing for cNTnC a rigid and open orientation (Ferrières et al., 2000). Additionally, this interaction was disrupted by the phosphorylation of Ser23/24 during the β-adrenergic stimulus to induce a lusitropic condition. More interesting, when the R145G cTnI inhibitory peptide mutant was incorporated for the MD simulations, the cNTnI_1–41_-cTnI_128–147_ interaction did not occur, and this extreme N-terminal region of cTnI maintained contact with the cNTnC region regardless of the presence of phosphomimetic mutations. In the same line, the R146G (R145G in human) and R21C cTnI mutants altered the PKA-dependent effects on weakening cTnC-cTnI interactions and accelerating myofibril relaxation (Cheng et al., 2015). These observations explain how R145G reduces the modulation of the cTn complex by S23/24 phosphorylation upon β-adrenergic stimulus and adds evidence for intramolecular contact in cTnI triggered by phosphorylation (Lindert et al., 2015).

The C-terminal end segment of cTnI is the most conserved structure among the cTnI isoform and species and is the site of the G203S and K206Q mutations. This region presents several charged amino acids and a highly flexible structure and seems to interact with Tm, playing a role in the Ca^2+^ switch of the thin filament (Sheng and Jin, 2014). Both mutants affect the backbone structure of cTnI; in particular, K206Q increases the maximum levels of ATPase activity and the filament sliding velocity (Deng et al., 2003; Köhler et al., 2003). Moreover, G203S disrupts the interaction between cTnT and cTnC, resulting in Ca^2+^ deregulation. Transgenic mice expressing the cTnI mutant G203S revealed a faster inactivation rate of the L-type Ca^2+^ channels and a greater increase in the mitochondrial membrane potential and metabolic activity upon activation compared to wt myocytes (Tsoutsman et al., 2006; Viola et al., 2016).

Remarkably, there is only one identified mutation in the N-terminal region of cTnI so far: the R21C mutant (Gomes et al., 2005). This mutation is located within the consensus phosphorylation site of PKA. *In vitro* studies demonstrated that R21C increases the Ca^2+^ sensitivity but reduced the phosphorylation levels when incubated with PKA. The physiological effect of PKA on decreasing the Ca^2+^ sensitivity of force development was diminished in cTnI R21C (Gomes et al., 2005). Additionally, the generation of R21C knock-in mice revealed the interesting behavior of this intriguing mutant. Top-down electron capture dissociation mass spectrometry revealed that R21C indeed depleted the phosphorylation status of S23/S24 cTnI in R21C homozygous (+/+) mice and decreased it by 8% in R21C heterozygous (+/−) mice compared to wt mice, supporting R21 as a crucial residue for PKA recognition and subsequent phosphorylation (Wang et al., 2012). Additionally, heterozygous R21C mice incorporated ~25% of R21C to the thin filament. The development of an HCM phenotype with cardiac hypertrophy, fibrosis, and the activation of the fetal gene program in both +/+ and +/− R21C mice supports a negative-dominant effect of this pathogenic mutant to the wt cTnI that is intensified to diastolic dysfunction and excitation-contraction uncoupling upon long-term ablation of cTnI phosphorylation (Dweck et al., 2014). Of note, these mice (+/+) also presented distinct contractile forces when comparing left and right ventricles (Liang et al., 2015). Curiously, the use of post-mortem heart tissues revealed ~56 and 1% cTnI phosphorylation when comparing normal and affected patients, respectively. Thus, mapping cTnI phosphorylation levels presents a promising biomarker for the early detection of hypertrophy (Zhang et al., 2011).

## Troponin T

cTnT is the subunit responsible for anchoring the cTnC and the cTnI to the thin filament and serves as an important communication switch in transferring the conformational changes induced by Ca^2+^ over the cTn complex and Tm (Leavis and Gergely, 1984; Tobacman, 1996). Due to genetic shuffling, several TnT isoforms are expressed across species, cell types and within the cellular environment (Anderson et al., 1991; Perry, 1998). The main structural difference between cardiac and skeletal TnT is the length of the N-terminal domain segment. This region carries the major structural multiplicities that are generated by genetic shuffling and is known as the hypervariable region. During heart development, an alternative splicing of exons 4 and 5 gives rise to different variants in size, physicochemical features and, thus, functions and Ca^2+^ responsiveness (Anderson et al., 1991; McAuliffe and Robbins, 1991). Four TnT isoforms can be detected in the heart: TnT1 (all exons), TnT2 (exon 4 is spliced out), TnT3 (exon 5 is spliced out), and TnT4 (exons 4 and 5 are spliced out) (Gomes et al., 2002). Due to this arrangement, the TnT molecular weight varies from 31 to 36 kDa, presenting 250–300 amino acid residues (Perry, 1998). In addition, altered patterns of cTnT expression were observed in heart failure and hypertrophy. Protein levels are directly associated with disease severity, suggesting an important role in the pathological state (Anderson et al., 1991, 1995; Townsend et al., 1995; Saba et al., 1996). The TnT is classically divided in two domains, T1 and T2, based on observations by Ohtsuki (1979). The proteolytic cleavage of skeletal TnT generates two different fragments, both binding to Tm. The T1 segment corresponds to the N-terminal region and comprises the hypervariable and central regions (Potter et al., 1995; Oliveira et al., 2000; Gollapudi et al., 2013). The hypervariable region plays a regulatory role in the Tm-binding affinity of TnT site 1 (Amarasinghe and Jin, 2015). The central region is highly conserved across species, playing a key role in anchoring the Tn complex to the thin filament through strong interaction with Tm and performing multiple functions (Heeley et al., 1987; Lehrer and Geeves, 1998; Palm et al., 2001; Regnier et al., 2002; Tobacman et al., 2002; Hinkle and Tobacman, 2003). The C-terminal T2 region interacts with TnC, TnI, actin and a second Tm binding site (Perry, 1998; Jin and Chong, 2010). *In vitro* studies have reported that TnT presents several phosphorylation sites, including Thr197, Ser201, Thr206, and Thr287 (mouse sequence) (Noland et al., 1989; Wei and Jin, 2011). Upon phosphorylation, these regions seem to be negative regulators of maximal force and Ca^2+^ sensitivity. In particular, Thr206 phosphorylation is critical for the functional properties of the thin filament (Sumandea et al., 2003, 2009). Although phosphorylation seems to play an important regulatory role in cTnT, the same pattern of phosphorylation was not observed *in vivo*. Of note, cTnT from mouse, rat and human tissues appears to be either monophosphorylated in Ser2 or unphosphorylated (Perry, 1998; Sancho Solis et al., 2008; Zhang et al., 2011; Streng et al., 2013). One reasonable explanation for these discrepant phosphorylation results is the high degree of N-terminal region conservation compared to other phosphorylation sites. In addition, the tridimensional arrangement of cTnT in the thin filament could occlude the other phosphorylation sites. Indeed, the specific role of Ser2 phosphorylation remains unclear (Solaro and Kobayashi, 2011). Monasky and coworkers revealed in a mouse model that the p21 kinase regulates cTnT phosphorylation in global myocardial ischemia and reperfusion injury. This result suggests that cTnT phosphorylation regulates cardiac homeostasis (Monasky et al., 2012; Streng et al., 2013).

Inherited cardiomyopathies caused by cTnT mutations account for ~15–30% of all HCM reports. In this context, the two cTnT-Tm anchoring regions are particularly interesting, harboring the vast majority of mutations observed so far (Willott et al., 2010; Figure 3). Once again, we would like to stress that the maintenance of key intermolecular interactions in this complex system is particularly important for the homeostasis of the thin filament function. Thierfelder and coauthors reported the first cTnT variations associated with HCM (Thierfelder et al., 1994). cTnT mutations spread in an autosomal dominant manner and appear to develop a malignant effect with a high incidence of sudden death (Watkins et al., 1995). Several mutations increase the Ca^2+^ sensitivity of force development and the force-pCa relation, e.g., I79N, R92Q, R92L, R92W, R94L, and A104V. However, the maximum force generation, ATPase activity and Ca^2+^ cooperativity are maintained. On the other hand, R278C decreases the Ca^2+^ cooperativity of force generation in skinned fibers, in addition to the Ca^2+^-sensitizing effect (Morimoto et al., 1998, 1999; Morimoto, 2007). These defects illustrate that cTnT mutations may alter not only the Ca^2+^ affinity for the myofilament but also intermolecular contacts. Mutations in position 92 show different effects on the folding of the cTnT tail domain (Hinkle and Tobacman, 2003). Additionally, interactions of R92Q, R92W, R92L, and R94L with Tm-dependent functions were reported to be impaired (Palm et al., 2001). An interesting atomistic model has been proposed to identify how these mutations affect allosteric modulations through the thin filament (Manning et al., 2012). More interesting, R92L, R92W, and R94L are still able to induce the muscle generation of force in a Ca^2+^-dependent manner, even under an acidic pH. The resistance to pH also suggests a role in the poor prognosis of HCM (Harada and Potter, 2004). A contraction event at low pH (e.g., ischemia) would decrease the intracellular levels of ATP, causing an up-regulation of the cytokines involved in the activation of apoptotic pathways (Morimoto et al., 1998). Structurally, these mutations promote an increase in cTnT helical stability, suggesting a more rigid structure (Palm et al., 2001). Moreover, the measure of Tm affinity decreased in all cTnT mutants, strongly suggesting that the supposed disordered Tm anchoring region plays a role in Tm-cTnT intermolecular contacts (Palm et al., 2001; Manning et al., 2012). cTnT mutations tend to be clustered in a conserved region comprising residues 92 (R92Q, R92L, R92W) and 160–163 (D160E, E163R, and E163K). Positions 160–163 are located within the conserved, highly charged region (158-RREEEENRR-166) and due to their flexibility are believed to play an important role in the regulation of the thin filament. An interesting study coupling *in vitro* and *in vivo* studies revealed that this region is exposed to the solvent. Mutations in this cluster alter critical electrostatic interactions for proper allosteric communication that leads to the transition from the blocked to the closed state (Moore et al., 2013, 2014). When expressed in mouse hearts, structural changes induced by R92Q were able to increase ATP consumption in the intact beating heart. However, using activating Ca^2+^ concentrations, R92Q decreases the energy-driven force, leading to a failure in the contractile performance (Tian and Ingwall, 1996; Chandra et al., 2001; Javadpour et al., 2003; Schwartz and Mercadier, 2003; Jimenez and Tardiff, 2011). This evidence suggests that this mutation is able to disrupt the myofilament Ca^2+^ sensibility probably due to impaired Tm-cTnT interactions (Takahashi-Yanaga et al., 2001b).

Another arginine replacement was reported in position 278 located on the C-terminal end of cTnT (Yanaga et al., 1999). When reconstituted in rabbit cardiac myofibrils (Sirenko et al., 2006) or skinned cardiac muscle fibers (Yanaga et al., 1999), R278C shows an increase in the Ca^2+^ sensitivity of ATPase activity; however, the maximum force cooperativity decreases. Moreover, R278C is able to disorder the α-helical structure of the wt cTn in addition to modifying the interface between the cTn core and the rest of the thin filament (Sirenko et al., 2006). In transgenic mice carrying R278C, the effect of Ca^2+^ sensitivity was not observed; however, the decrease in the maximal force corroborates the results of previous studies (Hernandez et al., 2005). cTnT-R278C impairs cardiac relaxation and diastolic function, which may be related not only to alterations in the cross-bridge cycling and/or detachment but also to alterations in Ca^2+^ sensitivities, contributing to the pathogenic effects of this mutant. Interestingly, Brunet and coworkers proposed a model to explain the structural and molecular role of both cTnT R278C and cTnI R145G. These mutations appear to affect the sliding event, indicating possible molecular explanations for the observed diastolic dysfunction (Brunet et al., 2014). Additionally, some cTnT mutations (e.g., R92Q and K280N) appear to be insensitive to the regulatory role of cTnI phosphorylation (Messer et al., 2016).

## Troponin C

Different TnC isoforms are expressed in human cardiac/slow skeletal and fast skeletal muscle cells and are encoded by the *TNNC1* and *TNNC2* genes, respectively. cTnC presents ~70% identity with the skeletal form. During evolution, the three-dimensional arrangements were widely conserved between both isoforms but diverged in their ability to bind Ca^2+^. cTnC plays an important role in the regulation of muscle contraction and relaxation due to the binding of Ca^2+^. TnC belongs to the superfamily of EF-hand proteins, comprising two globular domains connected by a central alpha helix (Herzberg and James, 1985; Sundaralingam et al., 1985). A canonical EF-hand motif is composed of two alpha-helices surrounding a loop segment that is responsible for divalent ion coordination. EF-loops are flexible and enriched with negatively charged amino acids, such as aspartic and glutamic acid. The basic coordination geometry comprises a pentagonal bipyramidal arrangement, in which seven chelating groups are responsible for ion connection. Importantly, this is the same arrangement observed in solution. The six chelating residues are classified first based on the linear position and second by aligning the geometric axis of a pentagonal bipyramid: 1 (+X), 3 (+Y), 5 (+Z), 7 (−Y), 9 (−X), and 12 (−Z). Of note, the carboxylate group in the side chain of the residue at the twelfth position (glutamic acid in ~92% of cases) provides a bidentate bond (Gifford et al., 2007). The C-terminal domain of TnC, also called the structural domain, is essential for the interaction with thin filaments and is able to bind Ca^2+^ with high affinity (~10^7^ M^−1^) and competitively bind Mg^2+^ (~10^3^ M^−1^). Although the cNTnC has two Ca^2+^ binding sites, site I is inactive due to several loop substitutions that impair Ca^2+^ coordination, for example, the inclusion of hydrophobic residues instead of charged residues in the +X and +Y positions (Gillis et al., 2007). Thus, contraction starts when Ca^2+^ binds to site II, conferring upon the N-terminal domain a regulatory role (Kobayashi and Solaro, 2005). It is known that the amount of hydrophobic exposure driven by Ca^2+^ binding at the N-terminal domain directly influences the strength of the Ca^2+^ signal transmitted through the thin filament (Li et al., 1999). Under physiological conditions, the binding of Ca^2+^ at the N-terminal domain is not sufficient to trigger an open cTnC state. To achieve an active state, two factors are important: (i) the binding of Ca^2+^ at site II and (ii) the interaction between cTnC and cTnI leading to changes in the number of hydrophobic patches and to the further stabilization of the open active state. Both factors are essential for contraction.

cTnC mutations associated with the HCM phenotype (Figure 3) alter two key mechanisms: the affinity for Ca^2+^ and the cTnC cellular partner interactions. Currently, the frequency of cTnC mutations is comparable to that of other targets, such as α-Tm and actin (Van Driest et al., 2003). The first described mutation was reported in a 60-year-old male patient presenting clinical signs of atrial fibrillation and hypertrophy of the left ventricle walls (Hoffmann et al., 2001). DNA sequencing revealed a T-to-A substitution at position 112, hence causing a leucine-to-glutamine exchange at amino acid 29. This mutation corresponds to the +X position within inactive site I; however, L29Q can change the Ca^2+^ sensitivity (Schmidtmann et al., 2005; Dweck et al., 2008; Liang et al., 2008; Neulen et al., 2009; Gollapudi and Chandra, 2012). Although divergent data exist with regard to whether this mutant leads to a decrease or increase in the cTnC Ca^2+^ affinity, the presence of Gln at position 29 may destabilize the A helix (Liang et al., 2008), thereby disturbing the Ca^2+^ binding properties at site II. Additionally, the A helix plays a role in the opening of cNTnC, an important stepwise mechanism for contraction (Li et al., 2000). Compared to wt, the L29Q mutant was insensitive to the interaction with a cTnI N-terminal region regardless of the presence of cTnI phosphorylation, suggesting desensitization to important biological contacts (Baryshnikova et al., 2008a; Li et al., 2013; Messer and Marston, 2014). Furthermore, using nuclear magnetic resonance to measure the N-terminal domain backbone dynamics of salmonid orthologous cTnC (ScNTnC), site I was more flexible than site II. ScNTnC displays a Gln at position 29 and results in a more open structure and a larger solvent-accessible area (Blumenschein et al., 2004). Finally, structural and functional assays revealed that the overall structure of L29Q has not changed, however small conformational dynamics were observed (Robertson et al., 2015). Based on the physiological mechanism of cTnC, these features help to understand the alterations in Ca^2+^ sensibility and protein-protein interactions. Another mutation located within site I was reported in a 5-year-old boy (Parvatiyar et al., 2012). The alanine exchange at position 31 to a serine was reported as a *de novo* mutation and introduces a polar amino acid into the Y position. This alanine is a highly conserved residue among different species, and the impact of this mutation results in severe alterations in the Ca^2+^ binding properties. Functional studies have revealed that A31S increases the Ca^2+^ affinity in isolated cTnC or in thin filaments. Moreover, A31S increases actomyosin ATPase activation and enhances thin filament activation (Parvatiyar et al., 2012). Mutagenesis and three-dimensional visualization showed that a hydroxyl group promotes an additional hydrogen bond with D33, thus providing rigidity to site I in a similar conformation as that for the skeletal TnC after Ca^2+^ binding (Parvatiyar et al., 2012).

Four other mutations associated with HCM phenotype were described, i.e., A8V, C84Y, E134D, and D145E (Landstrom et al., 2008). Functional studies have revealed that the E134D mutation has very similar Ca^2+^-binding affinities and force development as those of the wt (Pinto et al., 2009). Thus, this variant does not appear to be pathogenic, at least regarding these studied parameters. However, C84Y showed an increase in the Ca^2+^ sensitivity of force development and force recovery, in addition a sensitized ATPase activity of reconstituted myofilaments (Pinto et al., 2009). The A8V mutant is located in the *N*-helix, a region that plays an important role in the Ca^2+^ affinity of the N-terminal domain and interacts with helices A and D through hydrophobic and electrostatic interactions in the presence of Ca^2+^ (Herzberg and James, 1988; Gagné et al., 1995; Slupsky and Sykes, 1995; Houdusse et al., 1997; Strynadka et al., 1997). This *N*-helix mutant showed an increased Ca^2+^ sensitivity of force development and force recovery but did not appear to affect the intrinsic Ca^2+^ binding property (Landstrom et al., 2008). However, in the presence of the thin filament, A8V revealed an increase in the Ca^2+^ sensitivity of the reconstituted myofilament (Pinto et al., 2009, 2011a). Accordingly, A8V significantly increased the sensitivity of actomyosin ATPase regardless of the phosphorylation status of cTnI. In addition, A8V mutation led to a slower rate of Ca^2+^ dissociation in the presence or absence of phosphorylated TnI (Albury et al., 2012). Thus, it is reasonable that A8V affects cTnI-cTnC interactions or promotes an imbalance in cross-bridges that may further increase the Ca^2+^ affinity. Martins and coworkers reported the first animal model carrying the A8V heterozygous mutation. In agreement with *in vitro* studies, A8V revealed a Ca^2+^ sensitizer effect in mice. A8V mice developed right ventricular hypertrophy, hyperdynamic systolic function, atrial enlargement, fibrosis, and myofibrillar disarray, consistent with an HCM phenotype. Additionally, Ca^2+^ mishandling contributed to an altered contraction, leading to a severe heart remodeling. A reduction of phosphorylated cTnI (cTnI-P) levels was observed in A8V heart samples. This finding agrees with recent investigations suggesting that in humans, sarcomeric mutations have decreased the levels of cTnI-P (Sequeira et al., 2013; Martins et al., 2015). Finally, a molecular mechanism based on altered interactions between cTnC and cTnI has been proposed as the primary source of functional changes observed for myofilaments carrying the A8V mutation (Zot et al., 2016). The D145E mutant also revealed Ca^2+^ sensitizer effects in the myofilament. Interestingly, D145E is located at the +Z position within site IV and is the only mutation presented in the C-terminal domain that displays Ca^2+^-binding disarray. Steady-state fluorescence studies using IAANS revealed that the N-domain of D145E has a higher affinity for Ca^2+^ than does the wt in an isolated system or within the troponin complex (Pinto et al., 2011a). However, the ability to bind Ca^2+^ in the C-terminal domain appears to be drastically reduced (Swindle and Tikunova, 2010).

## Tropomyosin

Tm constitutes a diverse family of proteins that are ubiquitously expressed in eukaryotic cells. Four genes (*TPM1, 2, 3*, and *4*) integrate this multigene family in vertebrates, and each gene can produce different splicing isoforms in specific tissues. Of note, α-Tm is the predominant isoform expressed in the heart and comprises 284 residues. Gene shuffling leads to the expression of at least 40 Tm isoforms, and this multiplicity seems to play a pivotal role in cell maintenance (Gunning et al., 2005). Accordingly, using transgenic mice overexpressing β-Tm in the heart, Palmiter and coauthors showed that the replacement of α-Tm with β-Tm alters its structural and functional properties, leading to abnormal thin filament activation (Palmiter et al., 1996). Moreover, α-Tm plays a central role in regulating actin-myosin interactions that is indirectly controlled by the levels of Ca^2+^. A three-state model (blocked, closed, and open) has been proposed in an attempt to explain this dynamic and allosteric actomyosin regulation. Under resting conditions and low Ca^2+^ levels, the regulatory site of TnC is empty, characterizing the blocked state. Additionally, the interaction between actin and TnI is stabilized, and Tm locks actin-myosin interactions. The closed state is characterized by the binding of Ca^2+^ to the regulatory domain of cTnC and the exposure of a hydrophobic patch that further interacts with the C-terminal domain of cTnI. Moreover, the inhibitory peptide of cTnI moves away from actin. This transition state is characterized by the partial exposure of the myosin binding sites triggered by an azimuthal motion of Tm over the actin filament, generating weakly bound cross-bridges (Geeves and Lehrer, 1994; Lehrer, 1994). Finally, the open state is achieved in the presence of myosin heads, leading to the attachment of strong cross-bridges, allowing Tm to shift further on the actin filament and potentiate thin filament activation. Altogether, a tightly allosteric regulation through dynamic interactions is required to accommodate Tm along with the thin filament (Gordon et al., 2000; Kobayashi and Solaro, 2005; Solaro, 2010).

The human α-Tm is a coiled-coil dimer rolling over seven continuously actin monomers that provides actin filament support and the anchoring of the troponin complex. The α-Tm primary sequence consists of a short range of seven-residue pseudo repeats called a “heptad.” These residues are categorized as the form “*a-b-c-d-e-f-g*,” in which the *a* and *d* positions are often occupied by non-polar residues. These residues are responsible for the coiled-coil interface of the interaction and are essential for α-Tm stability. In contrast, the *e* and *g* positions present hydrophilic amino acids responsible for inter-helical salt bridges. The *b, c*, and *f* positions are exposed to the surface of the coiled-coil domain and are mainly filled with negatively charged residues that interact with the positively charged groove of F-actin (Wolska and Wieczorek, 2003; Barua, 2013; von der Ecken et al., 2015).

Similar to other sarcomeric genes, *TPM* gene mutations are also correlated with hypertrophic and dilated cardiomyopathy phenotypes (Thierfelder et al., 1994; Olson et al., 2001) but occur at very low frequencies (around less than 1%), as reported by large-scale studies (Richard et al., 2003; Van Driest et al., 2003). The incorporation of specific α-Tm mutants (A63V, K70T, D175N, and E180G) into adult cardiomyocytes revealed different isometric force measurements at submaximal Ca^2+^ concentrations, suggesting that HCM-related α-Tm mutants would predict clinical severity (Michele et al., 1999). Mutant transgenic mice were generated to investigate the pathological alterations triggered by the α-Tm D175N mutant, resulting in a severe impairment of heart contractility, relaxation and increased thin filament activation (Muthuchamy et al., 1999). Most of the HCM-related mutations in α-Tm increase thin filament Ca^2+^ sensitivity of force generation (Redwood and Robinson, 2013). However, different Ca^2+^ sensitivity measurements were obtained for D175N when considering transfected cardiomyocytes (mutant with a similar behavior to that of wt) and skinned fibers (higher Ca^2+^ sensitivity for the mutant). Interestingly, an evaluation of biopsies from two patients carrying the HCM-related α-Tm mutant D175N revealed the *in vivo* incorporation of the mutation within the *vastus lateralis* muscle, resulting in an altered contractile function (Bottinelli et al., 1998). The mutant and wt forms were shown to be equally expressed in this biopsies, demonstrating a negative-dominant profile for α-Tm in human muscle cells (Bottinelli et al., 1998). Following this study, different studies using reconstituted thin filaments with a wt/mutant mixture or Tm heterodimers were conducted with different results (Lakdawala et al., 2010; Janco et al., 2012). Of note, the *in vitro* production of α-Tm heterodimers carrying wt/D175N and wt/E180G revealed that mutations have little effect on dimer assembly and actin affinity compared to wt homodimers (wt/wt), but mutant homodimers have a slightly slower affinity compared to wt (Janco et al., 2012). More interesting, the D175N mutation was recently characterized using cardiomyocytes derived from patient-specific human-induced pluripotent stem cells (hiPSCs), in which D175N-hiPSCs revealed abnormal Ca^2+^ transients and prolonged action potentials compared to hiPSCs carrying the myosin-binding protein C Q1061X mutation (Ojala et al., 2016).

Recent studies have focused on the biochemical and biophysical characterization of HCM- and DCM-associated α-Tm mutations as a strategy to better understand the primary effects and consequences triggered by mutations in the long-range communication of the thin filament and specific phenotypes (Chang et al., 2014; Gupte et al., 2015). Additionally, the phosphorylation status of α-Tm and its effects on hypertrophic hearts were recently explored (Schulz et al., 2012, 2013; Schulz and Wieczorek, 2013). Although α-Tm phosphorylation dates back to the eighties (Ribolow and Barany, 1977), the protective link to hypertrophic phenotypes is just now emerging. Transgenic mice carrying the S283A α-Tm mutation that abrogates the α-Tm phosphorylation site exhibit a hypertrophic phenotype and increased protein levels and activity of the Ca^2+^ ATPase 2a (Serca) but, surprisingly, no changes in the myofilament Ca^2+^ sensitivity or the response to β-adrenergic challenges (Schulz et al., 2012). Accordingly, in a double-mutant transgenic mouse carrying the HCM-associated α-Tm mutant E180G together with S283A, the pathogenic phenotype of hypertrophic hearts was abrogated (Schulz et al., 2013).

Although several studies have had significant contributions, the impact of α-Tm mutations and phosphorylation on the mechanism developed by the protein during excitation-contraction coupling and its correlation with the hypertrophic phenotype remain speculative. Because this coiled-coil complex influences thin and thick filament interactions, we believe from a simplistic viewpoint that alterations in α-Tm would transmit structural changes by allostery on both sides of these contractile units. Therefore, further studies should explore the long-term effects of α-Tm mutations for a clear-cut correlation between the mutagenic profile and the hypertrophic phenotype.

## Allosteric communication defines the hierarchy of functional and pathological states

More than 80% of eukaryotic proteins share in their primary sequences intrinsically disordered regions (IDRs) flanked by packed domains. The complexity of these multi-domain architectures was in the past attributed only to their folded modules, but recent investigations and emerging techniques in structural biology have shown the key participation of IDRs as dynamic elements triggering signaling hierarchy and tuning protein functionalities. This new “*apple of the eyes*” has the potential to dissect hidden molecular mechanisms participating in physiological and pathological phenotypes. The premise that function follows structure has been left behind. A particular example is subunit I of the troponin complex (Hoffman and Sykes, 2008; Julien et al., 2010). TnI has IDRs with degrees of conformational flexibility that directly impact biological effects, mostly by changing the conformation and function of its partner, the TnC. The degree of disorder in the cTn complex was recently linked to HCM and DCM-causing mutations. Mutations mostly cause decrease in the disorder of cTnI and cTnT instead of an increase (Na et al., 2016). The structure and dynamics of the N-terminal region of cTnI were also explored, revealing the multiplicity of structural profiles assumed upon binding to the cNTnC (Hwang et al., 2014). Additionally, clear evidence of allostery inward of the troponin complex was revealed using pathogenic troponin T mutations. A mechanism in which changes in one protein indirectly affect a third through dynamic changes in a second protein reflects the allosteric transfer of information that culminates in pathogenic phenotypes (Williams et al., 2016). Moreover, the recovery of thin filament sliding speed of the double mutant cTnI-R145G-cTnT-R278C in comparison with cTnI-R145G alone provide further evidence of allosteric transmission within the Tn complex (Brunet et al., 2014). These allosteric mechanisms may represent an interesting strategy in future pipelines for therapeutic intervention of cardiomyopathies based on distant drugable sites inward of Tn subunits or even in other components of the sarcomere. The binding of different protein modules, recently characterized as supra-domain units (Papaleo et al., 2016), with different times, cellular conditions, and environments provides a new molecular repertoire for tuning communication transfer and is just now emerging.

The assembly of macromolecular complexes, such as the thin and thick filaments involved in muscle contraction, requires a well-tuned hierarchy of events for proper function. Regardless of the importance of this molecular motor in triggering human mobility, power stroke and heartbeat, correct communication among its individual elements is defined by allostery. Noteworthy is the fact that the switch-on and -off of this complex machinery is triggered by the influx and binding of Ca^2+^ to the C subunit of the troponin complex. The linker connecting the TnC structural and regulatory domains was shown to communicate both regions in a synergistic way (Grabarek et al., 1986; Moncrieffe et al., 1999; de Oliveira et al., 2013). The identification and functional characterization of HCM and DCM mutations in cTnC (Hoffmann et al., 2001; Landstrom et al., 2008; Willott et al., 2010) and other sarcomeric proteins (Geisterfer-Lowrance et al., 1990; Thierfelder et al., 1994; Redwood and Robinson, 2013; Chang et al., 2014), the alterations in Ca^2+^ sensitivity of force development and ATPase activity (Pinto et al., 2009, 2011a,b; Parvatiyar et al., 2012), and the appearance of the disease phenotype point toward a multidirectional change of allosteric pathways throughout the entire filament. Of note, an HCM-causing mutant at the Ca^2+^-binding site IV of cTnC (D145E) leads to an increased affinity of the Ca^2+^-binding site II (Pinto et al., 2009), providing clear evidence for allosteric communications between both domains. The mechanism under which this mutation affects a distal region of cTnC is still unclear but probably altered dynamics at the structural cTnC domain due to ion impairment at site IV, and communication transfer through the N-/C- linker might occur (Swindle and Tikunova, 2010). In agreement with this hypothesis, an H/D exchange analysis of wt cTnC revealed unprotected behavior in residues linking both domains, suggesting a higher mobility of this segment (Kowlessur and Tobacman, 2010). A short-term intra allosteric communication of D145E would impose molecular recognition changes in cTnC biological partners that ultimately lead to the HCM phenotype. Extensively dynamic propagation to cTnI inhibitory regions may also occur through the release of Ca^2+^, thus revealing dynamic adjustments throughout the entire troponin complex (Kowlessur and Tobacman, 2012).

Personalized structural and functional studies of hypertrophic and dilated cardiomyopathy-related mutants provide a framework to start assessing the plethora of short- and long-term allosteric pathways and to unveil the mechanisms behind their pathogenic effects. For example, the L29Q cTnC mutation involved in HCM does not drastically affect protein dynamics but reveals a slight increase in backbone flexibility at the cTnC regulatory domain (Robertson et al., 2015). Because intramolecular dynamics do not explain L29Q effects, changes in the long-term molecular recognition of its biological partners should justify its pathogenic behavior. Indeed, L29Q abolishes the effect of force-generation myosin cross-bridges (Robertson et al., 2015). In the case of A8V, a more open N-terminal domain conformation was observed compared to the wt in the apo and holo states, as revealed by paramagnetic relaxation enhancement (Cordina et al., 2013). In the dilated cardiomyopathy G159D mutation, no abrupt changes in backbone dynamics were observed by T1 and T2 relaxation rates. However, a weak anchoring of cTnI to this mutant was revealed by NMR chemical shifts and NOE connectivity patterns (Baryshnikova et al., 2008b), providing insight to explain the disease phenotype. This weaker interaction probably results in increased levels of acto-myosin inhibition and reduced ATPase activity (Mirza et al., 2005). Changes in allosteric communication are not exclusively related to disease-associated mutations but rather are also related to post-translational modifications. The N-terminal region of cTnI is sensitive to phosphorylation by PKA (Chandra et al., 1997) at specific serine residues (Ser23/24), which results in changes in the Ca^2+^ sensitivity of the cNTnC through cTnI itself and through interactions with cTnT (Wattanapermpool et al., 1995; Schmidtmann et al., 2002). The kinase promiscuity of Ser23/24 in cTnI (Solaro et al., 2013) reveals the convergence of multiple signaling pathways and the crucial role of cTnI as a central hub for communication transfer. The phosphorylation of cTnI by PKA enhances the rate of closing the cTnC N-terminal domain induced by Ca^2+^ dissociation compared to non-phosphorylated TnI, but this enhancement is abolished by both L29Q and G159D mutations (Dong et al., 2008), revealing a complex scenario of allosteric modulators. Regardless of intra- or inter allosteric changes triggered by cardiomyopathy mutations, the communication within this complex machinery is a highway containing several affluents, and an understanding of the short- and long-term dynamic maps would help to decipher the heterogeneous phenotype of cardiomyopathies and pinpoint new platforms for drug discovery.

## Structural biology efforts and treatment in the sarcomere

Structural biology is an exciting field that focuses on the elucidation of the three-dimensional architecture of biomolecules. This field dates back to the fifties with the breakthrough DNA model of Watson and Crick (Watson and Crick, 1953). The thin filament structure was first reported by Ebashi (1972). Groundwork using X-ray diffraction and electron microscopy provided the basis for Tn and Tm regulation in thin filaments (Huxley, 1972; Spudich et al., 1972; Parry and Squire, 1973). At the end of the nineties, the location of tropomyosin on F-actin filaments was determined from negatively stained electron micrographs (Lehman et al., 2000). Because Tn is repeated every seventh actin molecules, the density distribution of Tn is spread out during EM reconstructions. Thus, through a shorter Tn symmetry in thin filaments using an engineered internal deletion mutant of Tm, in which three of the seven actin-binding pseudo repeats were depleted, the Tn complex could be visualized by EM for three-dimensional reconstruction (Lehman et al., 2001). Considering Tn alone, some valuable efforts were made using X-ray diffraction (Takeda et al., 2003; Vinogradova et al., 2005), but because of limitations due to the flexibility and hydrophobicity of some terminal segments of TnI and TnT molecules, the entire Tn complex (~80 kD) could not be fully crystallized. Of note, half of the inhibitory segment of TnI (TnI_127–148_) is not observed in current crystal structures (PDB codes 1J1E and 4Y99), limiting the structural and functional characterization of this inhibitory segment for peptide assays (Lindhout and Sykes, 2003). Crystal structures of different constituents of the thin filament to align EM images were used to better represent this molecular assembly (Pirani et al., 2006; Poole et al., 2006). A single-particle analysis can help to better understand the structural complexity of the thin filament (Paul et al., 2009; Yang et al., 2014). Of note, cryo-EM structures were used to explain possible mechanisms during transition from the close to the open state of the actin:tropomyosin complex (Sousa et al., 2013). The correct orientation of Tn in the acto-Tm complex is a matter of debate. However, a recent application of single-particle procedures for molecular reconstructions has revealed the orientation of troponin on native relaxed cardiac muscle at a resolution of 25 Å (Yang et al., 2014). Regarding the question of thin and thick filament interactions, a recent electron cryomicroscopy structure provided a greater understanding of the human actomyosin complex at 3.9 Å, in which the F-actin myosin interface is stabilized by hydrophobic interactions throughout most of the interface (von der Ecken et al., 2016). The use of crystal structure comparisons and combined alignments provided structural details on actin-myosin conformational changes that discriminated the weak and strong myosin-binding states in F-actin. The authors declared that the lack information among states is due to the absence of intermediate conformations of myosin bound to F-actin, making interpretations of whether P_i_ is released before or after the powerstroke inconclusive (von der Ecken et al., 2015, 2016).

In the case of NMR, one tremendous advantage is the possibility to assess protein dynamics. Using ^1^H NMR spectra as a function of temperature, the groundwork from Wagner and Wüthrich revealed that aromatic side chains located inside a hydrophobic protein core may have rotational motions (Wagner and Wüthrich, 1978). The broad research community now accepts that molecular function is intimately related to dynamics. Several biological processes require dynamic transitional states, such as enzyme catalysis, in which key residues should be correctly positioned to coordinate the substrate at the active site, and ligand binding, which requires the entry of small molecules or ions to non-exposed clefts. Furthermore, in molecular recognition events and allostery, protein dynamics act as “*short-term memories*” to pass information. In addition, intra-motions and intermolecular motions that are transmitted to distal sites, also participate in the transfer of these “*memories*” for proper protein operation. NMR provides a unique opportunity to assess different biological motions ranging from picoseconds to seconds. For example, the use of the T1 and T2 relaxation rates is suitable for measuring fast dynamics on the order of a pico- to nanosecond timescale, while Carr-Purcell-Meiboom-Gill (CPMG) relaxation dispersion experiments are sensitive to millisecond motions (Carr and Purcell, 1954). Notwithstanding, CEST and DEST experiments are pushing toward the quantification of slow chemical exchange (Vallurupalli et al., 2012). Regardless of the size limitation for NMR studies, several of these experiments have been used to not only provide high-resolution NMR models but also to characterize the dynamics of cardiomyopathy-related mutants of isolated sarcomeric proteins. These data provide a unique strategy to start unveiling the communication transfer and allostery within the sarcomere.

NMR spectroscopy is also a very powerful technique for small-molecule screenings (Valente et al., 2006). Regarding this issue and focusing on sarcomeric proteins, TnC, the Ca^2+^ sensor of the sarcomere, has gained much attention. In addition to playing a crucial role triggering muscle contraction, TnC has become a feasible target for drug discovery because it is easily handled, and upon Ca^2+^ binding, TnC exposes “drugable” hydrophobic sites for small-molecule tests. The group of Dr. Brian Sykes has greatly contributed to identifying small-molecule candidates that bind to TnC (Hwang and Sykes, 2015). Both synthetic and natural molecules were studied by NMR and were shown to bind the regulatory and structural domains of TnC, e.g., trifluoperazine (Kleerekoper et al., 1998), bepridil (Li et al., 2000; Wang et al., 2002), levosimendan (Robertson et al., 2008), a W7 inhibitor (Hoffman et al., 2005; Hoffman and Sykes, 2009), EMD57033 (Wang et al., 2001), the flavonoid epigallocatechin gallate (Robertson et al., 2009), and the polyphenol resveratrol (Pineda-Sanabria et al., 2011). Most of these TnC binders act as Ca^2+^ sensitizers and bind to the TnC-TnI_148–163_ interface. Recently, a TnC-TnIchimera containing the switch segment of TnI (TnI_148–163_) was validated as a tool for producing isotopically labeled Tn peptides for NMR structural and drug-screening tests (Pineda-Sanabria et al., 2014). NMR spectroscopy was also used to characterize and validate a bifunctional rhodamine probe attached to cysteines of the skeletal TnC as a strategy for the *in situ* measurement of the orientation and motions of TnC (Mercier et al., 2003; Julien et al., 2008). Finally, the rational design of Ca^2+^-sensitizing mutants in the regulatory domain of cTnC is a good strategy for pinpointing key residues that would mimic the characteristic effects of cardiomyopathies and also to understand the properties of ion coordination and their structure-activity relationships (Tikunova and Davis, 2004; Parvatiyar et al., 2010).

While structural biology provides a mean to the understanding of how biomolecules behave in solution and has been used to uncover important molecular mechanisms within the sarcomere, cellular therapy, specifically the use of human pluripotent stem cell (hiPSCs), has the potential to reproduce physiological and pathological phenotypes *in vitro* (Han et al., 2014; Matsa et al., 2016). Viral transduction of combined transcription factors was able to reprogram fibroblats to pluripotent stem cells, opening up a tremendous advance for personalized medicine care (Takahashi and Yamanaka, 2006). The use of patient-specific cells for reprogramming technologies may work as platforms for the generation of a plethora of new differentiated cells, oriented drug discovery, toxicological studies, and new research-based investigations that mimic disease (Matsa et al., 2014; Guo et al., 2015). In HCM, for example, hiPSC-derived cardiomyocytes revealed hypertrophy, disorganization of the sarcomere and increased expression of genes, e.g., NFAT and calcineurin. More interesting, the use of calcineurin inhibitors have shown to reduce the hypertrophic phenotype (Lan et al., 2013).

## Signaling in hypertrophic cardiomyopathy

**Figure 4:**
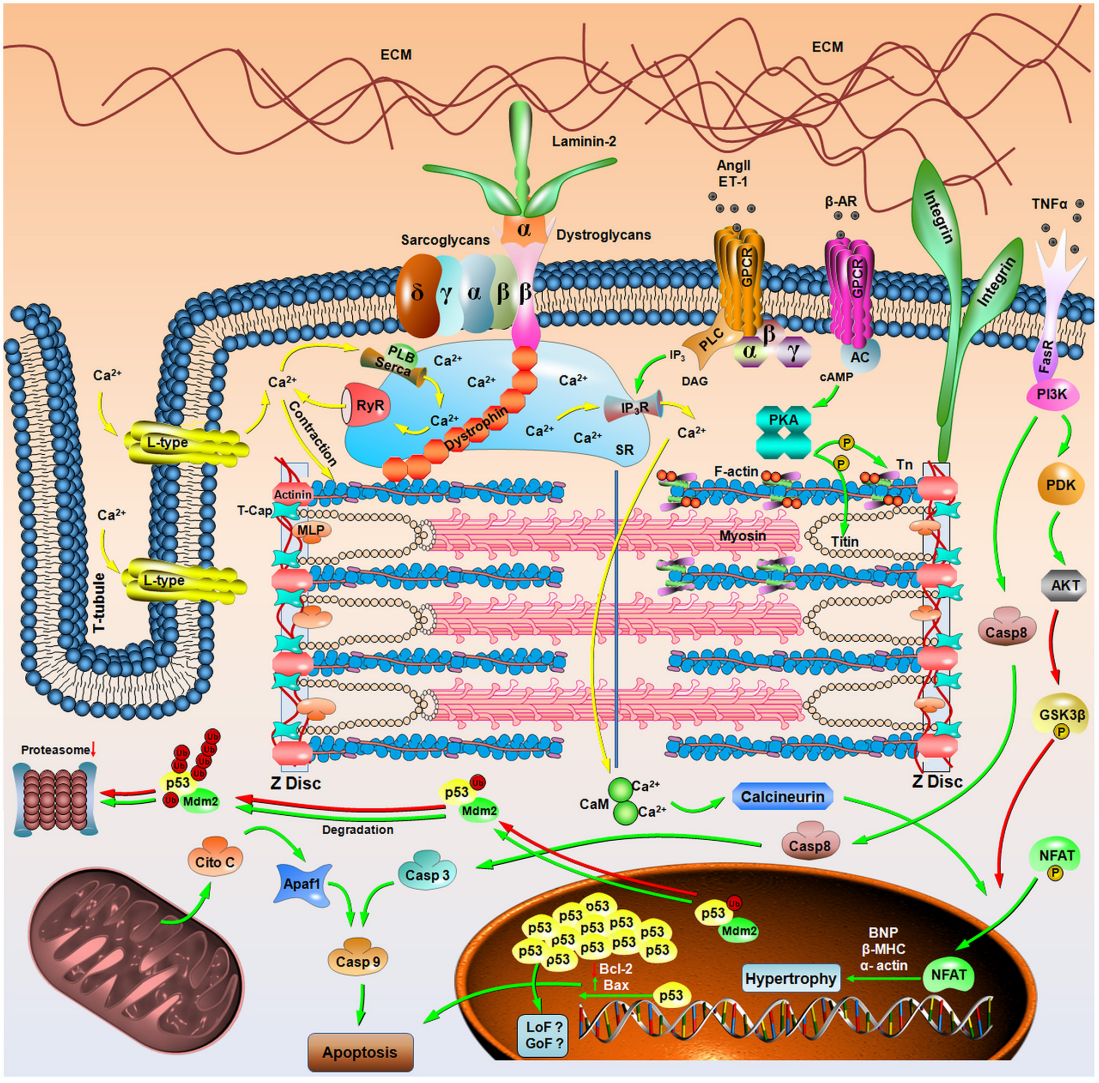
**Signaling in hypertrophic cardiomyopathy**. Schematic representation of the signaling pathways involved in hypertrophic cardiomyopathy. Green, red and yellow arrows represent promoting activity, inhibitory activity and signaling through Ca^2+^, respectively. “P” in yellow spheres and “Ub” in red spheres mean phosphorylation and ubiquitination. Molecules with abbreviated names are as follows: Cito C, cytochrome C; Casp, cysteine-aspartic acid protease; Apaf, apoptotic protease activating factor; NFAT, nuclear factor of activated T cells; BNP, brain natriuretic peptide; β-MHC, β-myosin heavy chain; CaM, calmodulin; MLP, muscle LIM protein; T-cap, telethonin; GSK3β, glycogen synthase kinase 3 beta; PDK, 3-phosphoinositide dependent protein kinase 1; PI3K, phosphoinositide 3-kinase; FasR, Fas receptor; TNFα, tumor necrosis factor α; GPCR, G protein-coupled receptor; β-AR, β-adrenergic; AngII, angiotensin II; ET-1, endothelin; AC, adenylyl cyclase; PKA, protein kinase A; PLC, phospholipase C; DAG, diacylglycerol; IP3, inositol 3-phosphate; IP3R, inositol 3-phosphate receptor; SR, sarcoplasmic reticulum; Tn, troponin complex; PLB, phospholamban; Serca, sarco/endoplasmic reticulum Ca^2+^-ATPase; RyR, ryanodine receptor; and ECM, extracellular matrix. LoF and GoF represent the loss-of-function or gain-of-function phenotype for p53.

Adult cardiomyocytes are differentiated and complex cells playing an unambiguous role in cardiac excitation-contraction coupling. This is achieved through a fine-tuned communication signaling of hundreds of molecules organized in weakly and tightly bound molecular assemblies, including cytosolic and transmembrane proteins, ion channels, transcriptional factors, adaptors, and second messengers (Figure 4). Unfortunately, this fundamental phenomenon is sometimes challenged by a repertoire of intrinsic and extrinsic insults or by the effects of sarcomeric mutations, the latter being the most common but not exclusive cause of cardiomyopathies. Due to disease complexity, the discussion of signaling changes will focus on HCM, where cardiac dysfunction is mainly credited to heart mass increase (hypertrophy), interventricular septal thickening, and fibrosis. The molecular events encompassing the HCM phenotype are increased protein synthesis, activation of genes expressed during embryogenesis, shifts in myosin isoforms, altered metabolism, and altered organization of the sarcomeric architecture.

Regardless of the sarcomeric repertoire of mutations causing HCM and heart tolerability to mechanical stretch upon heartbeat, the pathways discriminating physiological and pathological mechanosensitivities remain scarce. The correct tuning of biomechanical stress and how molecules sense and trigger hypertrophy is a matter of debate, but proteins located at the Z disc and at the costamere complex may play a role in this mechanotransduction process. The Z disc is responsible for transferring the tension between the sarcomeres through the interaction between titin and α-actinin (Luther and Squire, 2002). Other proteins were also identified in this environment, e.g., the muscle LIM protein (MLP) and telethonin (T-cap), in which mutations were already related to some cardiac diseases, including dilated cardiomyopathy (Arber et al., 1997). Costameres are complex protein assemblies connecting the sarcomere to the extracellular matrix and, together with the Z disc, act as a central station for sensing mechanostress and transmit information to alter contractile properties and transcriptional regulation to avoid heart failure. The mechanotransduction signaling triggered by these different elements aims to decrease blood volume, add sarcomeres to increase contractile capacity and change metabolism to favor energy production. Several lines of evidence link the mechanical signaling response to hypertrophic events, but for more in-depth information, please refer to more specialized literature (Sadoshima and Izumo, 1997 and references therein).

Heart hypertrophy is a compensatory event that is not exclusively related to pathology but rather is also related to the adaptation of the heart to altered contractility and biomechanical stress. Concentric hypertrophy mainly occurs due to pressure overload and is characterized by cardiomyocytes growing laterally with the parallel addition of sarcomeres. From the other side, excentric hypertrophy is due to volume overload, leading to longitudinal cellular growth and the “in-line” addition of sarcomeres (Dorn et al., 2003). Concentric hypertrophy in HCM is related to changes in cardiomyocyte alignment, while excentric hypertrophy is related to advanced HCM (Seidman and Seidman, 2001). One of the signaling pathways tuning the physiological (adaptation) or pathological response to hypertrophy is the phosphoinositide 3-kinase (PI3K)/serine-threonine kinase (Akt) cascade. Depending on the duration of Akt activation, different cellular responses take place, as observed from mice carrying ubiquitously activated Akt. The chronic activation of this kinase results in different phenotypes, ranging from moderate cardiac hypertrophy to massive cardiac dilation and sudden death (Matsui et al., 2002). In addition, Akt inhibits the downstream kinase glycogen synthase kinase 3 beta (GSK-3β) through phosphorylation. Once in the unphosphorylated state, active GSK-3β negatively regulates heart size from pathological insults (Haq et al., 2000; Michael et al., 2004). Transgenic mice expressing a TnT mutation involved in familiar HCM revealed that Akt activation and GSK-3β inactivation impact cardiac size and disease phenotype (Luckey et al., 2009).

Another important signaling triggered by pathological insults is the G-protein-coupled receptor (GPCR). These receptors are activated by factors released upon increased pressure or mechanical stretch, such as angiotensin II and endothelin (ET1), and play a role in hypertrophic responses. Additionally, GPCR-dependent PLCβ activation can lead to Ca^2+^ efflux from the sarcoplasmic reticulum and the activation of calmodulin. This downstream signaling activates the serine/threonine phosphatase calcineurin that dephosphorylates the nuclear factor of activated T cells (NFAT). Thus, NFAT translocates to the nucleus and reactivates the fetal gene program, which includes the expression of the brain natriuretic peptide (BNP), α-skeletal actin and β-myosin heavy chain (β-MHC) (Molkentin et al., 1998). The reactivation of cardiac fetal genes normally occurs in cardiomyopathies (Kuwahara et al., 2003), and these genes have been shown to be up-regulated in calcineurin transgenic mice (Molkentin et al., 1998), suggesting hypertrophic signaling as an alternative to overcome pathological effects. The increase in the β-MHC isoform and the concomitant decrease in the α-MHC isoform are great biomarkers for early cardiomyopathy (Lowes et al., 1997; Miyata et al., 2000).

The production and deposit of collagen type I and III by fibroblasts are other alterations observed in early cardiomyopathy characterizing fibrosis. Increased deposits may impair the excitation-contraction coupling, leading to severe changes in heart contractility (Menon et al., 2009). Hypertrophic cardiomyopathic hearts expressing gene mutations in α-myosin heavy chain were shown to increase the expression of TGF-β and stimulate non-myocyte cells to proliferate and express profibrotic molecules that ultimately lead to myocyte death, contributing to diastolic dysfunction in HCM hearts (Teekakirikul et al., 2010). The energy supply of cardiomyopathic hearts is also altered. Several changes occur to provide more ATP during the initial stages of cardiomyopathy, with fatty acid oxidation but shifts to glucose at advanced stages of the failing heard (Neubauer, 2007).

Several lines of evidence also link the activation of apoptotic signaling in hypertrophic hearts. An investigation of explanted failing hearts from transplanted patients with cardiomyopathy revealed increased levels of cytochrome C associated with the activation of the pro-apoptotic cysteine-aspartic acid protease 3, caspase-3 (Narula et al., 1999). Apoptotic activation was also observed in myocytes of transgenic mice overexpressing the Gs α-subunit coupled with GPC receptors upon the enhanced activation of β-adrenergic stimulus (Geng et al., 1999). Accordingly, upon norepinephrine stimulation, adult rat cardiac myocytes revealed apoptosis via PKA and voltage-dependent calcium channels (Communal et al., 1998). The increased mRNA and protein levels of the proinflammatory cytokine tumor necrosis factor-α (TNFα) in explanted hearts from dilated cardiomyopathic and ischemic patients and the TNFα-induced apoptosis in rat cardiomyocytes demonstrate the clear involvement of this programmed cell death mechanism in cardiomyopathy (Krown et al., 1996; Torre-Amione et al., 1996). Tumor suppressor p53 is also a key cell regulator and responds to DNA damage, inducing cell cycle arrest, senescence, DNA repair and apoptosis (de Oliveira et al., 2015). Interestingly, pacing-induced heart failure in dogs revealed increased protein levels of p53 with the downregulation of Bcl-2 and the upregulation of Bax in myocytes, consistent with an apoptotic scenario (Leri et al., 1998). A recent immunohistochemical study in HCM patients also linked increased levels of p53 and S100A4 proteins with an increased content of collagen fibers, thus, the modulation of these targets may ameliorate myocardial interstitial fibrosis (Qi et al., 2016). With regard to the growing literature on the involvement of aggregated forms of p53 in different cancer cells lines and tumor tissues (Levy et al., 2011; Ano Bom et al., 2012; Lasagna-Reeves et al., 2013; Silva et al., 2014; Yang-Hartwich et al., 2015) and given that p53 is marginally stable inside the cell and that aggregation depends on protein concentration, it is reasonable to argue that misfolded and aggregated forms of p53 might also occur in other unrelated diseases. Indeed, increased levels of endogenous p53 have already been reported in control and stressed myocytes, as well as during p53 overexpression (Miyashita and Reed, 1995; Pierzchalski et al., 1997; Leri et al., 1998). In addition, the short-lived p53 is targeted to the proteasome system due to the E3-ubiquitin ligase activity of its negative regulator, MDM-2. Of note, the decreased proteasome activity reported in HCM and failing human hearts (Predmore et al., 2010) supports the hypothesis of misfolded p53 accumulation and the formation of higher-order oligomers of this tumor suppressor. Furthermore, transgenic mice expressing a myosin-binding protein C mutation that were treated with a proteasome inhibitor were not able to regress the HCM phenotype but rather slightly improved cardiac function (Schlossarek et al., 2014). The loss-of-function and gain-of-function phenotypes of oligomeric and aggregated p53 and their involvement in the development of HCM are still matters of speculation and require further exploration.

## Concluding remarks

The combination of multidisciplinary expertise in structural biology, biochemistry, physiology, and cellular biology represent a *sine qua non* condition for better understanding the thin/thick filament interactions and ultimately the muscle contraction phenomena under normal and pathological conditions. The impact of mutations and post-translational modifications in sarcomeric proteins and their effects on the generation of non-adaptive hypertrophy are clarified from a series of well-designed studies. Because this review was written by structural biologists, the contribution that should come from this paper is the provision of a point-by-point investigation of the short- and long-term allosteric changes in different pathogenic mutations on isolated proteins and within the context of their biological partners. Because HCM presents a heterogeneous profile of genetic inherence, deciphering the altered motions and dynamic pathways through personalized mutagenic studies will certainly provide insight for new drugable sites that would have the potential for future therapeutics to hopefully ameliorate the hypertrophic phenotype from a broader range of pathogenic mutants.

## Author contributions

All authors listed, have made substantial, direct and intellectual contribution to the work, and approved it for publication.

### Conflict of interest statement

The authors declare that the research was conducted in the absence of any commercial or financial relationships that could be construed as a potential conflict of interest.
